# The impact of internal displacement on child mortality in post-earthquake Haiti: a difference-in-differences analysis

**DOI:** 10.1186/s12939-016-0403-z

**Published:** 2016-07-19

**Authors:** Bradley Chen, Timothy J. Halliday, Victoria Y. Fan

**Affiliations:** Institute of Public Health, National Yang-Ming University, 155 Linong Street, Sec. 2, Taipei, Taiwan 112; Department of Economics, University of Hawaii at Manoa, 2424 Maile Way, 533 Saunders Hall, Honolulu, HI 96822 USA; IZA, Bonn, Germany; University of Hawaii Economic Research Organization, Honolulu, HI USA; Department of Public Health Sciences & Epidemiology, University of Hawaii at Manoa, 1960 East–west Road, Biomed D204, Honolulu, HI USA; François-Xavier Bagnoud Center for Health and Human Rights, Harvard T.H. Chan School of Public Health, 651 Huntington Ave, Boston, MA USA; Center for Global Development, Washington, D.C., USA

**Keywords:** Infant mortality, Child mortality, Internally displaced people, Earthquake, Haiti

## Abstract

**Background:**

The Haiti earthquake in 2010 resulted in 1.5 million internally displaced people (IDP), yet little is known about the impact of displacement on health. In this study, we estimate the impact of displacement on infant and child mortality and key health-behavior mechanisms.

**Methods:**

We employ a difference-in-differences (DID) design with coarsened exact matching (CEM) to ensure comparability among groups with different displacement status using the 2012 Haiti Demographic and Health Survey (DHS). The participants are 21,417 births reported by a nationally representative sample of 14,287 women aged 15–49. The main independent variables are household displacement status which includes households living in camps, IDP households (not in camps), and households not displaced. The main outcomes are infant and child mortality; health status (height-for-age, anemia); uptake of public health interventions (bed net use, spraying against mosquitoes, and vaccinations); and other conditions (hunger; cholera).

**Results:**

Births from the camp households have higher infant mortality (OR = 2.34, 95 % CI 1.15 to 4.75) and child mortality (OR = 2.34, 95 % CI 1.10 to 5.00) than those in non-camp IDP households following the earthquake. These odds are higher despite better access to food, water, bed net use, mosquito spraying, and vaccines among camp households.

**Conclusions:**

IDP populations are heterogeneous and households that are displaced outside of camps may be self-selected or self-insured. Meanwhile, even households not displaced by a disaster may face challenges in access to basic necessities and health services. Efforts are needed to identify vulnerable populations to provide targeted assistance in post-disaster relief.

## Background

On January 12, 2010, an earthquake of magnitude 7.0 struck Haiti near its capital. The earthquake killed a total of 222,600 people and displaced 1.5 million people (IDP) [1–3]. Poor physical infrastructure in Haiti has been cited as a primary cause of the excess number of deaths from injuries in the disaster’s immediate aftermath [4]. In the medium to long-term aftermath, however, poor public health infrastructure has been cited as a primary cause of excess mortality [5], exacerbated by weak governance of the Haitian state [6]. Many IDP relocated to camps where anecdotal accounts described inadequate sanitation and security [3, 7]. Other IDP relocated to other areas in Haiti.

A few studies based on surveys have shown that the earthquakes had detrimental effects on household welfare. For example, Saint-Macary and Zanuso showed that households that were disproportionately impacted by the earthquakes had lower participation in labor markets [8]. They also found that households that received material assistance and/or moved to camps saw larger reductions in their assets. Similarly, Novella and Zanuso showed that households that were most affected by the earthquakes were less likely to invest in the human capital of their children [9].

In this paper, we contribute to this literature by estimating the impact of displacement on mortality and key mechanisms explaining health status. We utilize the Demographic and Health Survey from Haiti for 2012. Specifically, we estimate the causal impact of internal displacement to a camp or to a non-camp setting in another location within Haiti on child mortality. To address confounding variables that are related to both our outcomes and displacement, we employ matching methods in conjunction with a difference-in-differences (DID) research design. We also examine associations of key mechanisms explaining health status such as anemia and stunting and utilization such as immunization and bed net use.

## Methods

### Data and research design

Our primary data source to evaluate the impact of internal displacement on health is the fifth Demographic and Health Survey in Haiti [10]. The survey was conducted two years after the Haiti earthquake from January to June 2012. The information on children and birth history came from a nationally representative sample of 14,287 women aged 15–49. The standard core DHS questionnaire collects rich information regarding housing and socioeconomic status of households, health care utilization, and a number of maternal and child health outcomes including child mortality and biometric indicators.

As households and individuals that were internally displaced by the earthquake may be inherently different from those who were not, a simple comparison of health outcomes between the displaced and non-displaced would yield biased results which reflect the underlying drivers of their displacement. Therefore, we adopt a two-stage research design to enhance inter-group comparability. First, using coarsened exact matching, we match individuals on a number of household characteristics that would affect their likelihood of being displaced by the earthquake. Second, we assess the impact of internal displacement on infant and child mortality among those coming from similar households. This minimizes the effects from uncontrolled factors that affect both displacement and child health.

The 2012 Haiti survey asked questions especially pertaining to the earthquake. For instance, it asked individuals whether they “live in current housing during the earthquake,” based upon which we determined whether the individual had been displaced. In addition to standard household covariates, questions on earthquake-related housing damage and mortality were also used to match and enhance the comparability of households.

The key health outcomes in our study are infant mortality and under-five child mortality. The information is captured through the full birth history recalled by the interviewed women and recorded in the survey. To provide a more comprehensive picture of health status, health interventions and behaviors and to explore potential mechanisms affecting mortality, we further examined the relationship of displacement on key indicators of health status such as anthropometric growth (height for age) and anemia level (ordinal indicating severity), uptake of public health interventions of bed net use, spraying against mosquitoes, and vaccination for BCG, measles, and DPT, as well as conditions of experiencing hunger in the last 4 weeks, and infection with cholera after October 2010.

### Statistical analysis

#### Matching

Our analyses begin by matching individuals on household characteristics which delivers a similar likelihood of displacement across groups. We employ coarsened exact matching (CEM) for this purpose. Conceptually, CEM is similar to exact matching, but rather than requiring matched pairs to have exactly the same covariate values, they are matched by meaningful groups of covariate values [11]. We choose CEM over some “approximate matching” methods including propensity score matching (PSM) for several reasons. First, PSM may lead to a poorer imbalance on matched covariates, which in certain circumstances may compromise the validity of the research design, perhaps, more so than non-matching [12, 13]. In contrast, CEM belongs to monotonic imbalance bounding matching methods, which bound the maximal imbalance by *ex ante* choice [14]. Second, for approximate matching methods such as PSM, in addition to the iterative process of estimation, matching and balance checking, its application also requires analyses to be limited to “common support” so as to remove extrapolations beyond data limits and model dependency. CEM, on the other hand, limits the analyses to common empirical support automatically. There are a number of other benefits, described extensively in the literature [11, 13, 14].

We perform a logit regression to explore potential predictors of camp residence and displacement. Based on the results, we match households on their likelihood of being displaced based upon the following characteristics: location of residence (region, urban/rural), household size, gender of household head, wealth index, and whether the housing was damaged during the earthquake.

#### Difference-in-differences (DID)

The DHS records the full birth history of the interviewed women including births prior to the earthquake. This allows us to employ a DID design to identify the births prior to the earthquake as controls. We use a logit model to analyze the impact of IDP on infant and child mortality, with the following specification:

$$ {\mathrm{M}}_{it}=\alpha +\beta Lo{c}_i+\gamma Pos{t}_i+\delta \left(Lo{c}_i*Pos{t}_t\right)+\theta {X}_i+\eta {Z}_i+{\varepsilon}_{it} $$

where, *M*_*it*_ refers to the logit of the probability of the binary dependent variable indicating whether the live birth *i* born in year *t* died or not prior to 1 year of age (infant mortality), and whether a live birth died prior to reaching 5 years of age (child mortality). The variable *Loc*_*i*_ indicates the location of the household. Households belong to one of the following three groups: camp resident, displaced but not residing in a camp, and non-displaced (in the same housing after earthquake). The variable *Post*_t_ is a dummy variable indicating whether the birth was born after the earthquake. The DID interaction term between *Loc* and *Post* in our model and its coefficient, *δ*, captures the impact of displacement on infant and child mortality controlling for inherent mortality differences prior to the earthquake. The vector *X*_*i*_ is a set of household characteristics, such as geographical location, household size, household head characteristics, wealth and land ownership, and damage by the earthquake. We also control for a number of birth characteristics, *Z*_*i*_, which include indicators for a twin birth, gender, birth order, and preceding interval in months. The household and birth characteristics adjust for any remaining imbalance resulting from the coarseness in our matching procedure.

We next explore the key mechanisms explaining child mortality by estimating a logit model in the cross section. All the dependent variables are binary except for the time to water source (continuous), and the severity of anemia (ordinal—non-anemic, mild, moderate and severe), for which an ordinary least squares model and an ordered logit model are estimated respectively. Once again, we control for household and child characteristics.

## Results

### Descriptive statistics

Our investigation is based upon the matched sample of 21,417 births from 6011 households. Descriptive statistics on these matched births by displacement status are presented in Table 1. Approximately one-third of the matched births were from displaced households, with 9.69 % residing in camps and the rest living elsewhere. Prior to matching, the three groups were different (descriptive statistics of the pre-matched sample are presented in Appendix 1); CEM matching reduced this imbalance among households of different displacement statuses.

**Table 1:** Descriptive statistics of household characteristics and earthquake damage among matched birth

Variable	Non-displaced	Non-camp IDP	Camp IDP
Matched rate, % of sample	68.06 %	87.44 %	87.30 %
Number of households (% of matched households)	3,669 (61.03 %)	1,662 (27.65 %)	680 (11.31 %)
Number of births (% of total)	13,867 (64.75 %)	5,474 (25.56 %)	2,076 (9.69 %)
Household size, mean	5.20	5.44	4.35
Urban, %	48.13 %	42.44 %	63.15 %
Wealth Index, %			
1 = Poorest	15.09 %	20.81 %	0.00 %
2	14.79 %	19.86 %	1.45 %
3	37.42 %	21.17 %	80.25 %
4	21.11 %	22.62 %	17.15 %
5 = Richest	11.59 %	15.55 %	1.16 %
Land ownership	55.44 %	60.61 %	28.42 %
Household Head			
Gender: female, %	46.49 %	42.38 %	57.32 %
Age, mean	43.27	41.07	37.88
Highest education attained, secondary and higher, %	23.44 %	28.92 %	34.63 %
Impact of earthquake			
Housing damage by earthquake, %	57.58 %	46.22 %	87.52 %
Family member(s) killed, %	3.09 %	5.20 %	8.83 %

### Model analyses

Point estimates from our model showed that households residing in camps experienced increased infant and child mortality relative to households that were not displaced, whereas households that were displaced but not re-located to camps saw reductions. We also found that infant and child mortality was the highest in the camps, despite higher access to key mechanisms of health status compared to households elsewhere.

### Impact of displacement on infant and child mortality

The results of the DID analyses are shown in Table 2. Across the three models, post-earthquake births were associated with lower mortality compared to those pre-earthquake, e.g. the OR of post-earthquake was 0.49 (95 % CI 0.32 to 0.75) for infant mortality and 0.38 (95 % CI 0.24 to 0.58) for child mortality among IDP, indicating that child survival improved over time.

**Table 2:** Impact of displacement status on infant and child mortality

	Camp vs. Non-displaced (*n* = 10,261)		Camp vs. Non-camp IDP (*n* = 4,428)		Non-camp IDP vs. Non-displaced (*n* = 12,449)	
Dependent variable	OR	95 % CI	OR	95 % CI	OR	95 % CI
Infant mortality						
Unadjusted	0.57	0.17 to 1.85	2.54**	1.41 to 4.57	0.22**	0.08 to 0.65
Camp/Displacement*Post-earthquake						
Adjusted^a^						
Camp/Displacement	0.75	0.35 to 1.60	0.88	0.61 to 1.28	1.14	0.83 to 1.57
Post-earthquake	0.72	0.36 to 1.41	0.49***	0.32 to 0.75	0.73	0.37 to 1.43
Camp/Displacement*Post-earthquake	1.19	0.48 to 2.99	2.34*	1.15 to 4.75	0.58	0.27 to 1.26
Under-5 child mortality						
Unadjusted	0.57	0.19 to 1.67	2.37**	1.38 to 4.07	0.24**	0.09 to 0.67
Camp/Displacement*Post-earthquake						
Adjusted^a^						
Camp/Displacement	0.91	0.48 to 1.73	0.81	0.59 to 1.11	1.28	0.98 to 1.66
Post-earthquake	0.54*	0.30 to 0.96	0.38***	0.24 to 0.58	0.56*	0.31 to 1.00
Camp/Displacement*Post-earthquake	1.34	0.58 to 3.08	2.34*	1.10 to 5.00	0.63	0.31 to 1.27

***p < 0.001, **p < 0.01, *p < 0.05

^a^All adjusted models control for birth characteristics (twin birth, sex, preceding interval and birth order), region, location (rural/town/city), altitude, household size, sex, age and education level of the household head, wealth index, land ownership, whether housing was destroyed and family members killed by earthquake

Our DID captured differential trends in mortality. The estimates of the interaction term between displacement and post-earthquake in the fully adjusted model showed that births in camp households had higher infant and child mortality than the two other groups. After the earthquake, the camp IDP births compared to non-camp IDP births had a high OR = 2.34 (95 % CI 1.15 to 4.75) of infant mortality and OR = 2.34 (95 % CI 1.10 to 5.00) of child mortality. Odds ratios of camp births compared to births in non-displaced households were 1.19 (95 % CI 0.48 to 2.99) in infant mortality and 1.34 (95 % CI 0.58 to 3.08) in child mortality. This is consistent with the finding that the births from non-camp IDP households had lower infant and child mortality than the non-displaced births. Among these two groups, displacement was associated with odds ratios of 0.58 (95 % CI 0.27 to 1.26) and 0.63 (95 % CI 0.31 to 1.27), for infant and child mortality, respectively. Similarly, the unadjusted model also shows that non-camp IDP births had the lowest infant and child mortality among the three groups and that there is no statistically significant difference in mortality between births from camp and non-displacement households.

As a sensitivity analysis, we restricted the pre-earthquake control sample to those within 5 and 7 years before 2010 earthquake, respectively (Appendix 2). Estimates are consistent and showed that camp births had higher infant and child mortality than non-camp IDP births while their mortalities were not statistically different than those of the non-displaced ones.

### Health status and utilization

To explore mechanisms through which displacement status affects infant and child mortality, we examined the associations between displacement status and indicators of health status and uptake of public health interventions. Results are presented in Table 3.

**Table 3:** Association between displacement status and child health status and health behaviors in post-earthquake Haiti^a^

	Camp vs. Non-displaced			Camp vs. Non-camp IDP			Non-camp IDP vs. Non-displaced		
Dependent variable	N	OR	95 CI	N	OR	95 CI	N	OR	95 CI
Child health status									
Stunting	1,910	1.06	0.41 to 2.72	1,077	1.44	0.85 to 2.45	2,503	1.40	0.99 to 1.98
Anemia level	1,718	1.03	0.59 to 1.81	938	1.57*	1.07 to 2.30	2,248	0.94	0.76 to 1.17
Malaria									
Bed net use	4,083	3.23***	2.03 to 5.17	2,152	1.76***	1.29 to 2.42	5,051	1.39**	1.13 to 1.69
Indoor spraying	4,072	22.53***	11.08 to 45.80	2,138	11.07***	4.64 to 26.42	5,036	1.27	0.82 to 1.97
Vaccination^b^									
BCG vaccination	1,971	2.62*	1.18 to 5.81	1,157	0.90	0.56 to 1.43	2,534	1.56*	1.01 to 2.40
Measles vaccination	2,472	2.33*	1.16 to 4.65	1,407	0.91	0.61 to 1.37	3,179	1,23	0.91 to 1.67
DPT vaccination (3 shots)	2,478	1.43	0.77 to 2.65	1,406	0.86	0.56 to 1.32	3,184	0.96	0.71 to 1.30
Other conditions									
Hunger	4,084	0.58*	0.38 to 0.88	2,151	1.06	0.76 to 1.49	5,051	0.72***	0.59 to 0.87
Cholera after Oct 2010	4,084	0.55	0.30 to 1.01	2,152	0.64*	0.42 to 0.97	5,052	0.98	0.78 to 1.24

***p < 0.001, **p < 0.01, *p < 0.05

^a^ All models are adjusted for region, location (rural/town/city), altitude, household size, sex, age and education level of the household head, wealth index, land ownership, whether housing was destroyed and family members killed by earthquake. Stunting refers to height-for-age < −2SD

^b^Measles vaccination refers to aged 1 year or older. DPT vaccination refers to fully vaccinated (3 shots) for children aged 1 year or older. BCG vaccination refers to children born after the earthquake (Jan 2010). Analyses are limited to subgroups of children in the sample. For BCG vaccination, analyses are performed to those born after the earthquake as the vaccine is recommended at birth. For measles and DPT vaccines, the analyses are performed for children at least one years of age. For recommended vaccination schedule, please see World Health Organization (2015) [23]

We assessed two non-mortality biomarker-based health measures: growth for age and level of anemia. Our analyses detected a significantly higher likelihood of having severe anemia among children in camp households than those in the non-camp IDP households (OR = 1.57, 95 % CI 1.07 to 2.30). There was no significant difference among household of different displacement status in anthropometric growth, an indicator of long-term household stresses and chronic food insecurity.

Across the indicators on uptake of public health interventions, camp households consistently had relatively better access to basic necessities. Compared to non-displaced households, camp households were more likely to use a bed net for sleeping (OR = 3.23, 95 % CI 2.03 to 5.17) and to spray their households against mosquitoes (OR = 22.53, 95 % CI 11.08 to 45.80). Moreover, camp households also had better access to water, with a regression coefficient for displacement at −5.37 (95 % CI −9.41 to −1.32) (OLS results not shown). Non-camp IDP households also had significantly higher use of a bed net (OR = 1.39, 95 % CI 1.13 to 1.69) compared to the non-displaced households.

We further investigated differences in having received vaccines recommended during early childhood (specifically BCG, measles, and DPT). Compared to non-displaced children, children in camps were significantly more likely to have been vaccinated against measles by 1 year of age (OR = 2.33, 95 % CI 1.16 to 4.65) and, among children born after the earthquake, to have been given the BCG vaccine (OR = 2.62, 95 % CI 1.18 to 5.81). Children in non-camp IDP households were also more likely to have received the BCG vaccination than their counterparts in non-displaced households (OR = 1.56, 95 % CI 1.01 to 2.40).

Other dependent variables assessed were the experience of hunger in the last four weeks and cholera infection of any family member since October 2010. Compared to non-displaced households, camp households were less likely to have experienced hunger (OR = 0.58, 95 % CI 0.38 to 0.88) and were also less likely to have any family member that suffered from cholera since the inception of the cholera outbreak (OR = 0.55, 95 % CI 0.30 to 1.01). In contrast, non-camp IDP households were less likely to go hungry (OR = 0.72, 95 % CI 0.59 to 0.87) compared to non-displaced households.

## Discussion

The increasing frequency and intensity of natural disasters has wrought significant economic toll on populations around the world [15]. When coupled with poor infrastructure, these disasters become particularly devastating by displacing numerous people and producing complex social and health situations. In this study, we examined the impact of household displacement as a result of the 2010 Haiti earthquake on child health and explore potential underlying mechanisms. To our knowledge, our study is the only study which has used nationally representative data to quantitatively assess the impact of internal displacement by natural disasters on health.

There are several limitations to our study. Similar to all studies based upon surveys, our results are susceptible to recall bias by the interviewed mothers. This is true for birth histories that are further away from the time of the interview. Recall bias also likely occurred in the documentation of health behaviors and uptake of public health interventions (but not biomarker-based indicators). Unlike our analyses on infant and child mortality, our analyses on health behavior mechanisms used a cross-sectional design, preventing definitive conclusions about the directionality of potential causal relationships. For instance, our observation that camp households were less likely to have experienced hunger recently and yet their children were more likely to have severe anemia, could be due to households with poor food intake previously being drawn to camps since camps were thought to have adequate food supply from aid agencies and organizations.

Our record of the displacement status was limited to three categories (moved to a camp, moved elsewhere, or did not move) and lacks variability and precision of the intensity of displacement from one place to another. Hence, the variable did not allow us to assess the dose–response relationship of displacement on health, because the displacement status is determined by the information of residence at the time of the interview. We lack data on how long a household had been displaced or has been living in a camp, or how the housing quality changed from one location to another. Households reporting to reside in camps could have been displaced and lived in camps since the immediate aftermath of the earthquake or some time afterward. Likewise, non-camp IDP households may have never lived in a camp, or may have moved out of a camp by the time of the survey. Thus, the identified differences between the two displaced populations in our models may be under-estimated.

After accounting for socioeconomic factors that shape the susceptibility of households being displaced by the earthquake, we find that survival of children in camp households was most adversely affected by the earthquake, followed by those in households that were not displaced.

In contrast to our expectations, the households that were displaced but did not reside in camps seem to be least impacted in terms of children’s health. Although intuition might be that the displaced population was more vulnerable than the non-displaced one [3], the IDP was in fact heterogeneous. Households displaced *other* than to camps had greater reductions in infant and child mortality compared to those displaced to camps. These findings may partially be explained by selective migration. That is, people who moved in response to the earthquake could be self-selected as unobserved health problems may compromise mobility [16]. Alternatively, the migration of households could be a manifestation of self-insurance strategies with extended families or friends who can provide shelter in hardship, a common informal risk-coping strategy in developing countries [17]. This is consistent with our observation that the non-camp IDP households enjoy better access to resources which improve health. These findings highlight the importance of migration as a strategy to reduce the impact of a disaster [18].

There was no significant difference in child mortality between those displaced to camps and those not displaced. Compared to IDP groups, children in non-displaced households had poorer access to resources which can improve health. This implies that, for some households with the same likelihood of displacement, staying behind results in poorer living conditions and access to health services; they might live in a damaged house in a disaster-prone area but simply cannot afford or lack the networks to migrate. Future studies and investigations are required to understand the challenges and difficulties confronting these vulnerable households. Although they are not internally displaced, they are frequently overlooked. For governments and agencies working in post-disaster relief, beyond the displaced population, efforts need to be dedicated to identify and reach out to households that could be “left behind” in damaged areas.

Households in camps experienced higher infant and child mortality than households not displaced to camps, and our investigation into household conditions and health practices yielded additional insights into possible mechanisms. On one hand, households in camps seem to have good access to resources which can improve health such as food and water access. On the other hand, children in camps do suffer from a higher likelihood of severe anemia, which is consistent with their higher infant and child mortality. This raises the issue of the quality of food to enhance their physical well-being.

Without cause of death data, we can speculate on the potential reasons for differences in mortality observed. Infectious disease and malnutrition are well-known causes of child mortality in complex emergencies. Although efforts to vaccinate may reduce risk against certain diseases such as measles, mortality risks persist from risk factors associated with higher risk of diarrhea and respiratory infections. In particular, malnutrition (including protein energy malnutrition) resulting from food insecurity and limited access to nutritious food in camps can lead to compromised immunity and increased susceptibility to infections, resulting in further energy loss, and triggering a vicious cycle of deteriorating nutritional status and illness. We found higher rates of hunger and anemia of children in camps compared to those displaced elsewhere, despite higher rates of vaccination coverage.

A third explanatory factor may be the risks associated with heightened security risks of living in camps. News reports suggested an increased incidence of both sexual and other physical violence in camps, which in turn could lead to higher mortality and morbidity from physical injury, mental health, and challenges to caretakers and mothers in their ability to care for their children. Indeed, according to the 2012 Haiti DHS report, 36 % of women aged 15–49 living in camps reported to have experienced physical violence, and 16 % having experienced sexual violence, both higher than the population averages at 28 % and 13 %, respectively [10]. Prevalence of domestic violence in camps was also a notable issue in camps at 29 %.

Finally, our study’s emphasis on physical health should not de-emphasize the importance of mental health as an essential dimension of human health, especially in a complex emergency context.

## Conclusions

Our use of a DID design to evaluate the impact of displacement on infant and child mortality, as well as a series of examinations into the association between displacement status and access to resources which can improve health, underline complexities in post-disaster relief. Not only is it the case that there is much work to be done to improve the health of those living in camps and that these households are likely to face a wide range of short and long-term challenges in the years to come, the displacement population itself is heterogeneous. Moreover, even individuals that are not displaced by disasters are not well-protected and risk-free. Evidence-based and better-targeted interventions require greater knowledge and better systematic evaluations of what works and who is at risk. Given the extensive impact of the earthquake on Haiti, all public health interventions including those that tackle water treatment, vaccination, and vector control [19–21] should consider the post-earthquake housing context. Despite the US$6 billion in aid to Haiti, there are few evaluations of services delivered and lives saved [22]. Scholars, governments and international agencies should begin to systematically collect and analyze information on disaster impact, so that we can make progress to establish resilient and responsive health systems that can truly be the safety nets of our populations.

## Abbreviations

CEM, coarsened exact matching; DHS, Demographic and Health Survey; DID, difference-in-differences; IDP, internally displaced people; PSM, propensity score matching

## Appendix 1

**Table 4:** Descriptive statistics of the pre-matched sample

Variable	Non-displaced	Non-camp IDP	Camp IDP
Number of households (% of total)	5,008 (65.50 %)	1,871 (24.47 %)	767 (10.03 %)
Number of births (% of total)	20,375 (70.23 %)	6,260 (21.58 %)	2,378 (8.20 %)
Household size, mean	5.94	4.92	3.99
Urban, %	32.12 %	50.40 %	70.14 %
Wealth Index, %			
1 = Poorest	28.45 %	14.86 %	0.00 %
2	22.76 %	16.83 %	8.60 %
3	16.87 %	21.33 %	73.14 %
4	16.53 %	26.94 %	17.21 %
5 = Richest	15.38 %	20.15 %	1.04 %
Land ownership	72.34 %	55.21 %	25.55 %
Household Head			
Sex (female), %	39.36 %	43.99 %	58.02 %
Age, mean	45.25	38.75	35.78
Highest education attained, secondary and higher, %	24.56 %	39.76 %	41.85 %
Impact of earthquake			
Housing destroyed by earthquake, %	26.18 %	46.23 %	86.83 %
Family member(s) killed, %	1.00 %	5.36 %	9.28 %

## Appendix 2

**Table 5:** Sensitivity analyses limiting pre-earthquake control births within 5 and 7 years before the 2010 earthquake

	Camp vs. Non-displaced			Camp vs. Non-camp IDP			Non-camp IDP vs. Non-displaced		
	N	OR	95 % CI	N	OR	95 % CI	N	OR	95 % CI
Infant Mortality: Whether the children die by 12 months old									
All year included	10,261	1.19	0.48 to 2.99	4,428	2.34*	1.15 to 4.75	12,449	0.58	0.27 to 1.26
Excluding births prior to year 2005	3,655	0.93	0.32 to 2.69	1,826	2.43*	1.09 to 5.45	4,535	0.52	0.24 to 1.10
Excluding births prior to year 2003	4,641	0.99	0.33 to 2.98	2,257	2.52*	1.11 to 5.75	5,760	0.52	0.25 to 1.10
Child Mortality: Whether the children die by 5 years old									
All year included	10,261	1.34	0.58 to 3.08	4,428	2.34*	1.10 to 5.00	12,449	0.63	0.31 to 1.27
Excluding births prior to year 2005	3,655	0.83	0.30 to 2.31	1,826	2.00	0.84 to 4.76	4,535	0.55	0.28 to 1.09
Excluding births prior to year 2003	4,641	0.87	0.30 to 2.51	2,257	1.92	0.79 to 4.63	5,760	0.59	0.29 to 1.20

**p* < 0.05
